# CLIPPERs - Clip the right protein – a new tool for targeted proteolysis in bacteria

**DOI:** 10.1038/s44319-025-00532-3

**Published:** 2025-07-25

**Authors:** Marta Carroni

**Affiliations:** Science for Life Laboratory, Department of Biochemistry and Biophysics, Stockholm University, Stockholm, Sweden

**Keywords:** Methods & Resources, Microbiology, Virology & Host Pathogen Interaction, Post-translational Modifications & Proteolysis

## Abstract

CLIPPERs are a new variant of PROTAC degraders that are applicable to Gram-negative and Gram-positive bacteria.

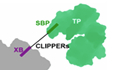

## Teaser

Despite antibiotics, bacterial infections still represent a major challenge to human health as bacteria have been developing resistance to most of the currently available antibiotics. Ideally, we need to develop not only new antibiotics but also explore novel strategies to fight pathogenic bacteria. Targeted protein degradation (TDP) as a method for fighting antibiotic-resistant bacteria has already been proposed for *Mycobacteria*. A new study in *EMBO reports* shows a novel variant of TPD that is applicable to both Gram-negative and Gram-positive bacteria, using the conserved AAA+ protease ClpXP (Izert-Nowakowska et al, 2025). This could potentially expand the toolkit for fighting antibiotic resistance to a broader class of pathogens.

**Figure 1:**
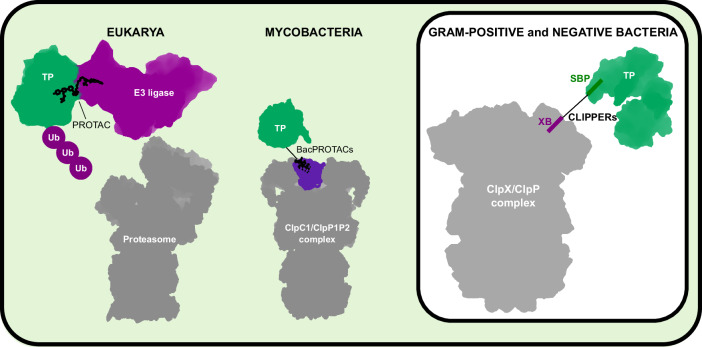
Schematic summary of TPD approaches in eukaryotes, mycobacteria (Actinobacteria), and in Gram-positive and Gram-negative bacteria (as in Izert-Nowakowska et al, 2025). TP targeted protein, SBP strong binding peptide, XB ClpX-recruiting peptide XB.

Traditional approaches to drug development, both to treat human diseases or to fight infectious microbes, work by inhibiting specific proteins in conserved molecular pathways. The strategy of targeted proteolysis, instead, works by depleting the target protein via the cell’s own protein-degradation system. A number of so-called PROTACs have been developed to activate the ubiquitin proteasome system in eukaryotic cells (Békés et al, 2022). PROTAC degraders bring their target protein into close proximity to the E3 ubiquitin ligase, which labels the protein for degradation (Fig. 1). More recent approaches work by escorting the protein close to the proteasome or by activating autophagy. This strategy allows to target proteins that are otherwise difficult to block using small molecules. Since their invention around 20 years ago, different variants of PROTACs have been designed (Békés et al, 2022), some of which are already being tested in the clinic (Chirnomas et al, 2023).

While PROTACs are based on eukaryotic proteolytic systems, the study in *EMBO reports* by Maria Górna and her team (Izert-Nowakowska et al, 2025), suggests that the concept of TPD could be extended to bacteria, which, like eukaryotes, possess dedicated and controlled systems for proteolysis to maintain proteostasis and cellular fitness (Izert et al, 2021). In fact, bacterial degradation tags (degrons) have been used for research purposes since the early 1990s, for instance, to control specific protein levels in *E. coli*.

In 2020, Gopal et al, 2020 found that the anti-tuberculosis drug pyrazinamide targets *M. tuberculosis’* aspartate decarboxylase PanD to the ClpC1/ClpP1P2 protein-degradation machinery, possibly by exposing PanD’s C-terminal degron. Although this finding has high potential for developing novel anti-bacterial compounds, it is very much limited to both the protein target and the pathogen, and crucially depends on small molecules.

Instead, Górna and colleagues suggested, in an earlier publication, the design of more generalized bacterial TPD degraders, similar to the eukaryotic PROTAC, to shift the focus to the protease machinery and place any target protein in close proximity (Izert et al, 2021). In particular, they suggested AAA+ proteases, such as Lon or those of the Clp family—the latter composed of an ATPase unfoldase ClpX, ClpA, or ClpC and a ClpP peptidase part—as candidate degraders, as these are present in a broad range of bacterial pathogens.

The specificity of these bacterial proteolytic complexes owes to their ATP-driven unfoldase component, which consists of either an individual (ClpX) or a pair (ClpA or ClpC) of AAA + ATP pockets. The unfoldases have a highly variable N-terminal domain involved in recognition of degrons, unfolded protein regions, and adapter partners, to carefully select the substrates to be destroyed (Mabanglo and Houry, 2022).

A first BacPROTAC was developed in 2022 to target protein substrates to the ClpC1/ClpP1P2 complex in *Mycobacterium* (Morreale et al, 2022) (Fig. 1). The first BacPROTACs were also based on a small molecule, the antimicrobial cyclomarin A (cymA), a cyclic peptide that binds to the *M. tuberculosis* ClpC1 N-terminal domain and strongly enhances its unfoldase activity (Maurer et al, 2019). These BacPROTACs are constructed of two moieties: one that binds to the target protein (e.g., a small binding partner or compound) and one, cymA and variants, that binds to and activates ClpC1 (Morreale 2022). A subsequent study presented a new approach: the Homo-BacPROTAC based on dimerized cyclomarins, where ClpC1 would be both the target and the protein-degradation machinery so as to destroy itself (Junk et al, 2024). Although these classes of BacPROTACs are interesting and promising, they are limited to *Mycobacteriales*.

The new study in *EMBO reports* (Izert-Nowakowska et al, 2025) describes how Górna and collaborators engineered a protein degrader for *E. coli* called Clp-interacting peptidic protein erasers (CLIPPERs). CLIPPERs are based on the ClpX/ClpP protease complex, a highly conserved AAA+ protease in both Gram-positive and Gram-negative bacteria (Fig. 1). They consist of a linker that connects a peptide-binding part that targets the protein of interest—the chaperone GroEL for this proof-of-concept—and the XB peptide, which binds and activates ClpX. The ClpX-recruiting peptide XB was derived from SspB, a ClpX partner.

The best peptide binders to GroEL were based on the “strong binding peptide” (SBP), which was identified by phage display (Chen and Sigler, 1999); the same technique could be used to find selective and efficient binders to any protein of interest. GroEL-targeting CLIPPERs, so-called GroTACs, were tested for cell viability at different temperatures, followed by quantitative mass spectrometry to analyze both the expression of the GroTAC itself and the effect on the entire proteome, as it depletes a fundamental chaperone such as GroEL. The choice of GroEL as target could be though considered suboptimal, owing to the large phenotypical defects related to its depletion and thus the difficulty in pinpointing the exact chain of events in the TPD process. However, it is very interesting that major chaperone systems involved in protein homeostasis can be targeted for proteolysis: being so central for cell health, these can be attractive options for TPD antibiotic development.

The development of efficient and specific bacterial protein degraders is a promising approach for developing urgently needed new antibiotics, even if it is still in its infancy. The design of binders to recognize specific target proteins remains one of the main challenges, as the choice of what proteins to target so as to achieve the most efficient anti-bacterial effect. Moreover, the CLIPPER degraders in the highlighted study were expressed using an inducible plasmid. This is very useful for biotechnological applications, but it remains to be seen how to deliver these small peptides in a clinical setting.

This work shows that TPD of a major chaperone system can be an efficient strategy for developing novel antibiotics, given that even a little activation of the degrader will create major proteome misfunctioning. Many chaperones appeared downregulated upon GroTAC expression, but others, such as trigger factor, showed increased levels, suggesting the existence of a possible rescue mechanism (Izert-Nowakowska et al, 2025). This raises the question about how efficiently and fast we need to develop such bacterial degraders to avoid the activation of resistance mechanisms by the pathogen. Nonetheless, CLIPPERs constitute a substantial advancement and potentially a novel therapeutic approach applicable to the large majority of bacterial pathogens.
